# A Bibliometric and Visual Analysis of Single Nucleotide Polymorphism Studies in Depression

**DOI:** 10.2174/1570159X21666230815125430

**Published:** 2023-08-15

**Authors:** Zi Zhang, Ye Yang, Wan Kong, Shanqing Huang, Yaqian Tan, Shanshan Huang, Ming Zhang, Haoyang Lu, Yuhua Li, Xiaolin Li, Shujing Liu, Yuguan Wen, Dewei Shang

**Affiliations:** 1 Department of Pharmacy, The Affiliated Brain Hospital of Guangzhou Medical University, Guangzhou 510370, China

**Keywords:** Depression, SNP, bibliometrics, genes, citespace, polymorphism

## Abstract

**Background:**

Genetic polymorphism has been proven to have an important association with depression, which can influence the risk of developing depression, the efficacy of medications, and adverse effects *via* metabolic and neurological pathways. Nonetheless, aspects of the association between single nucleotide polymorphisms and depression have not been systematically investigated by bibliometric analysis.

**Objective:**

The aim of this study was to analyze the current status and trends of single nucleotide polymorphism research on depression through bibliometric and visual analysis.

**Methods:**

The Web of Science Core Collection was used to retrieve 10,043 articles that were published between 1998 and 2021. CiteSpace (6.1 R4) was used to perform collaborative network analysis, co-citation analysis, co-occurrence analysis, and citation burst detection.

**Results:**

The most productive and co-cited journals were the Journal of Affective Disorders and Biological Psychiatry, respectively, and an analysis of the references showed that the most recent research focused on the largest thematic cluster, “5-HT”, reflecting the important research base in this area. “*CYP2D6*” has been in the spotlight since its emergence in 2009 and has become a research hotspot since its outbreak in 2019. However, “*BDNF* ”, “*COMT* ”, “older adults”, “loci”, and “DNA methylation” are also the new frontier of research, and some of them are currently in the process of exploration.

**Conclusion:**

These findings offer a useful perspective on existing research and potential future approaches in the study of the association between single nucleotide polymorphisms and depression, which may assist researchers in selecting appropriate collaborators or journals.

## 1 INTRODUCTION

According to the World Health Organization (WHO), depression currently has a global prevalence of approximately 5% [1] and is one of the most widespread psychiatric disorders that severely affect human life [2, 3], and it affects approximately 4% of the global population [4]. Gender, genetics, environmental variables, and psychological factors are frequently linked to the pathophysiology of depression. According to certain studies, genetic variables are crucial in research on depression [5, 6]. People between the ages of 15 and 30 have a high prevalence of depression [7, 8]. The high mortality rate and high suicide risk caused by the increased prevalence of depression have a huge impact on society and also affect individual mood and behavior, compromising their ability to work, study, and live normally. Although depression is a treatable disease, there are vast individual differences in the efficacy of drug treatment [9], so only a handful of patients exhibit a satisfactory therapeutic effect [10]. Individual differences in drug reactions are predominantly affected by height, weight, age, sex, complicating diseases, disease progression, environmental factors, and genotype. Studies have shown that genetic polymorphisms explain about 42% of the individual variation in antidepressant response [11].

In the field of pharmacogenomics, different individuals show individual differences in efficacy, adverse reactions, and metabolic enzyme activities for different drugs due to genetic polymorphisms [12], and these differences are mainly due to the influence of genomic biomarkers, such as drug-related metabolic enzymes and transporters, on drug pharmacokinetics [13]. The real difference among individuals lies in the presence of single nucleotide polymorphism (SNP) [14], which occurs when one nucleotide is replaced by any of the other three nucleotides, resulting in a polymorphism [15]. In recent years, individualized treatment has received increasing attention, and interest in the association between SNP and depression has been maintained at a high level, as evidenced by a large number of research studies and reviews on this topic. For example, six SNPs in the tryptophan hydroxylase gene (*TPH1*, *TPH2*) have been studied to verify the association between polymorphisms in the *TPH* and the development of depression, and the tryptophan hydroxylase pathway is implicated in the pathogenesis of depression [16]. The glucocorticoid receptor (GR) gene is deemed a risk factor for depression. A study on the SNPs of NR3C1 in the Polish population found that the heritability and variation of this gene were associated with recurrent depression and speculated that the SNPs of this gene may be associated with the etiology of recurrent depression [17]. Other studies have similarly analyzed the correlation between gene polymorphisms and the disease process, etiology, and mechanism of various psychiatric and neurological diseases, such as depression [18], schizophrenia [19], and Alzheimer's disease [20]. While these research studies offer preliminary insight into the link between SNP and depression, the existing literature lacks a more comprehensive bibliometric analysis of this issue. Therefore, our study aimed to address this research gap in the literature.

The term “bibliometric”, defined as “the quantitative analysis of the bibliographic characteristics of the body of literature”, represents the emergence of the need for a new approach to structural knowledge and the ability to analyze a large number of publications at two different levels, micro and macro, while maintaining domain independence [21, 22]. The inclusion of temporal and spatial dimensions in the analysis distinguishes bibliometrics from other review analyses, providing new insights into the development and scholarship of a specific field [23]. This study used CiteSpace software to conduct a bibliometric analysis. CiteSpace is a software program designed by professor Chen Chaomei based on the JAVA application for visual analysis of literature trends [24, 25]. It extracts distribution-related information by analyzing different categories of indicators, such as countries/regions, institutions, journals, authors, keywords, and references, and identifies research hotspots and frontiers in a specific field in a short time [26]. After more than a decade of development, bibliometrics is now commonly used worldwide in medical research. These results will facilitate a timely review and analysis of research hotspots and trends in the area of SNP and depression and encourage the development of this field, while also promoting the etiology, prevention, and treatment of depression [27].

Consequently, this study attempted to comprehensively analyze the research status and development trend in SNP and depression from 1998 to 2021 by conducting a bibliometric and visual analysis.

## 2 MATERIALS AND METHODS

**Fig. (1):**
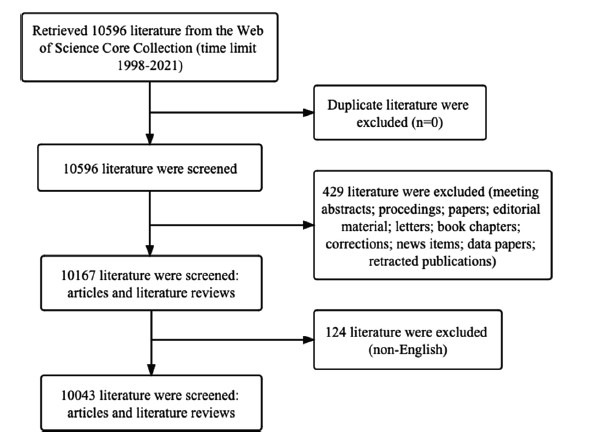
Flow chart of literature search.

This study referred to the Web of Science Core Collection (WoSCC) database, which is a subscription-based online science citation index service on the Web of Science^TM^ (WoS) platform (http://www.webofknowledge.com/). Compared to other databases, the WoS database contains more scientific publications and is a comprehensive data source for bibliometric software [28, 29]. As the database connection was kept open, a literature search was performed on this database on July 20^th^, 2022, to avoid deviations from daily database updates. The search strategy involved entering “depression,” “single nucleotide polymorphism,” and their synonyms into the search box to obtain the final search formula as follows: TS = (depression OR depressed OR depressions OR depressive OR despondent OR gloomy) AND TS = (SNP OR (single nucleotide polymorphism) OR (Single Nuclear Tide Polymorphisms) OR (single nucleotide polymo) OR (Haplotype-tagging SNPs) OR (gene polymorphism) OR (genotype) OR (polymorphism genetics) OR (genetic polymorphism)). In this study, the inclusion and exclusion criteria were as follows:

1) The time frame was from 1998 to 2021, covering a total of 23 years.

2) Other types of documents were excluded (*e.g*., letters, meetings, abstracts, clinical trials, books, news, and retracted publications) and only articles and reviews were kept.

3) No species limits were set.

4) Duplicate literature was eliminated.

5) Only literature published in the English language was included (Fig. **1**).

The functions of publication output, subject category, H-index, and impact factor used in the WoSCC literature analysis were used for the literature analysis. The analysis tool used in this study was CiteSpace (6.1 R4), which conducted a collaborative network analysis of the authors, countries/ regions, and institutions, co-citation analysis of journals, authors, and references, keyword co-occurrence analysis, and citation outbreak detection of keywords and literature. Parameter settings included the time slicing (1998-2021), node types, and selection criteria from each slice. The analysis results obtained by CiteSpace can be used to generate visualizations consisting of nodes and connecting lines. The types of research, such as authors, institutions, keywords, and countries/regions, are represented by each node in the graph, while the cooperation, co-reference, or co-occurrence between different nodes is represented by the lines between different nodes. In the network of CiteSpace, node size represents the number of published articles, the connection between nodes represents the cooperation and connection between different countries/regions, the color of nodes represents the time of publication, and the purple annual rings represent centrality 0.1.

## 3 RESULTS

### 3.1 Analysis of Publication Outputs and Growth Trend Prediction

**Fig. (2):**
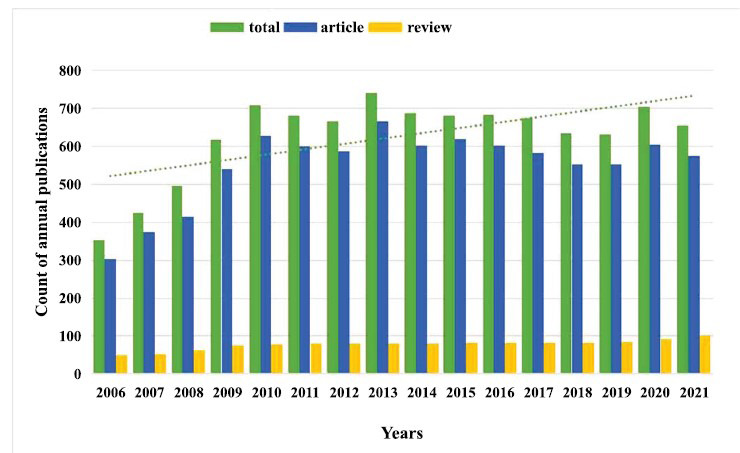
The number of publications from 2006 to 2021 on SNP research in the depression field.

According to the search results in the WOS, a total of 10,596 publications met the inclusion and exclusion criteria, including 8,805 and 1,238 articles and reviews. The growth of publications in this field can be divided into three stages: no articles dated before 2006 were found; a growth phase was then observed from 2006 to 2010; and this was followed by a relatively flat phase after 2010. The number of publications produced grew from 352 in 2006 to 741 in 2013 and remained above 600 from 2013 to 2021 (Fig. **2**). The average annual publication volume from 2006 to 2021 was 436, indicating a high publication rate in this field. According to the results obtained from searching the concept of “single nucleotide polymorphism”, we discovered that the documented literature on depression and SNP started in 2000, and an explosive increase was observed from 2006. A high level of research popularity occurred in 2010, which was consistent with the results of the study. The cause of depression's pathogenesis may be unknown [30, 31], and with the rapid development of modern society and an increasing number of cases of depression due to individual differences in patients and an increasing demand for individualized treatments of depression [32, 33], the research field of SNP and depression has maintained a relatively stable development trend [34].

### 3.2 Analysis of Scientific Collaboration Networks

According to the three levels of scientific cooperation network analysis provided by CiteSpace, we conducted an institutional and national cooperation network analysis of the literature. The collaboration network map between countries/regions has 130 nodes and 1,112 connections, as shown in Fig. (**3A**). SNP studies on different aspects of depression have been published in 130 countries. The top 15 countries/regions are listed by the count of publications, as shown in Table **1**. In terms of the count of publications, the United States ranked first with more than 3,000 publications (24.03% of the total), followed by England, Germany, China, and Australia with more than 4,000 publications (28.46% of the total).

From 1998 to 2021, a total of 587 institutions published research in this field. Table **1** lists the top 15 institutions based on publications. The top three institutions in this field with the highest contributions were King’s College London in the United Kingdom (314), the University of Toronto in Canada (213), and Duke University (198) in the United States. Seven of the top 15 institutions were located in the United States, whose higher scientific standing is reflected in its ranking as a country or area. The findings from the map indicated that countries conducting SNP research on depression worked closely together. The majority of the top 15 nations and organizations were developed nations. This may be because developed countries are more advanced in medical and scientific research and have more funds to invest in scientific research. Second, despite a large number of patients, research progress in developing countries may be relatively slow [35]. The generated institutional network map identified 586 nodes and 5,164 lines, which, respectively, represented the institutions and their cooperative relationships, thus highlighting extensive cooperation between institutions, as shown in Fig. (**3B**).

A total of 836 authors have published in the field over the past dozen years. Table **1** lists the top 15 authors with the highest number of publications. Six authors have published more than 50 papers in this field. With 150 articles, Serretti Alessandro leads the group of authors who are currently active in the field, followed by Rietschel Marcella and Domschke Katharina. The co-authorship network map is depicted in Fig. (**3C**) and has 3,489 cooperation lines connecting 836 nodes, which reflect the authors and their relationships of co-authorship. The figure shows that the authors work closely together and that there is no zonal cluster distribution. Aside from a small group of authors, the majority of authors have numerous intimate relationships and exchanges.

### 3.3 Analysis of Journals and Co-cited Journals

The articles included have been published in 1,354 different journals, many of them in specialized journals. Table **2** shows the top 15 journals and co-citation journals for SNPs in the depression field. The most prolific journal in this field is the Journal of Affective Disorders (324, 3.23%), followed by *Plos One* (283, 2.82%), and the American Journal of Medical Genetics Part B Neuropsychiatric Genetics (234, 2.33%). All high-yield journals had an IF score of 3.0 or higher, and all had more than 80 publications between them. The top 3 journals based on total citations are Biological Psychiatry (5,470), Molecular Psychiatry (5,249), and Archives of General Psychiatry (4,634).

### 3.4 Analysis of Co-cited Authors

A total of 1,329 authors were cited in the co-citation analysis of authors, 20 of whom were cited at least 300 times. The top 10 co-cited authors are listed in Table **3** along with their nations, affiliations, primary research areas, and H-index. A trustworthy reference indicator for evaluating each scientist's unique contributions to science is the H-index [36]. With 1385 citations, Caspi Avshalom led the field, followed by Lesch Klaus-Peter (1,611) and Kendler Kennet S (1,045), with the remaining authors receiving fewer than 1,000 citations. Kessler Ronald C has the greatest H-index value (227), followed by Kendler Kennet S (156) and Caspi Avshalom (149). Neuroscience, genetics, psychology, and pharmacology are their main fields of study. The foundation for and contributions to later research and advancement in this field have been laid by these authors.

### 3.5 Analysis of Co-cited References

Citations are frequently regarded as the core of bibliometric investigations. The articles in this field that have received the most co-citations typically represent essential basic research. The co-citation network is separated into 84 clusters, only 11 of which are the largest, as seen in the clustering visualization given in Fig. (**4**). GWAS (cluster #0), *5-HTTLPR* (cluster #1), pharmacogenetics (cluster #2), *BDNF* (cluster #3), *FKBP5* (cluster #4), inbreeding (cluster #5), tagging SNP (cluster #6), *TPH2* (cluster #7), *CYP2C19* (cluster #8), *HTR1A* (cluster #9), and ventral striatum (cluster #11) are among the various clusters represented in the graphic. The colors of the various clusters indicate the authors and the typical year of publication of the cited references, which are represented by the various nodes in the map. The average profile of each cluster is greater than 0.5, and the q-value is 0.7395, indicating that the clusters are of reasonable quality. The cited references and the relationships between them are represented by the 761 nodes and 4,421 links of the clustering visualization graph of the included reference co-citation network. Recent research has concentrated on cluster #8, “*CYP2C19*”, as seen in Fig. (**4**) (the mean year 2015). The top-ranked reference in Table **4**, which lists the top 15 citations for SNP research on depression, was written by Caspi Avshalom and cited 575 citations. According to the above study, Caspi Avshalom was also the most-cited author.

### 3.6 Analysis of Keywords

In the context of bibliometric analysis, keywords reflect hotspots and trends in a certain field of study and primarily reflect the article's or author's major points of interest. Based on the time zone view (Fig. **5**) of the keyword co-occurrence network to map the knowledge structure of research, 843 nodes and 10,911 links were produced, respectively, representing the keywords and their co-occurrence relationship. The largest node was “depression”, and the larger the node, the more frequently the keyword appeared. Table **5** shows the top 10 keywords, the most frequent of which was “depression”, followed by “polymorphism”, “association”, “major depression”, and “gene.” Additionally, since 2006, the terms “polymorphism” and “gene” have grown to be some of the most popular study topics, with 2,069 and 908 referenced studies, respectively.

**Fig. (3):**
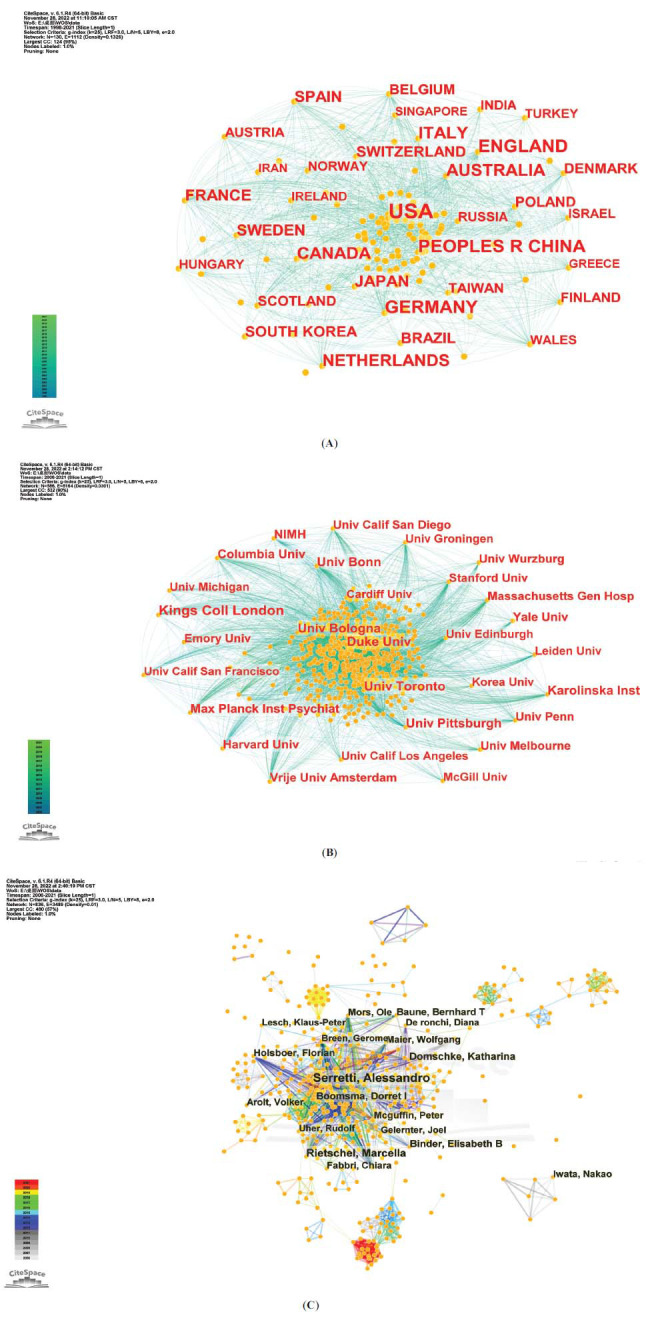
Visualization of the scientific collaboration network analysis for SNP research in depression 1998-2021. Collaborations between nations/regions (**A**), institutions (**B**), and authors (**C**). The map's nodes represent various components, including authors, nations/regions, and institutes, while the connecting lines between nodes show relationships among the participants in the project.

**Fig. (5):**
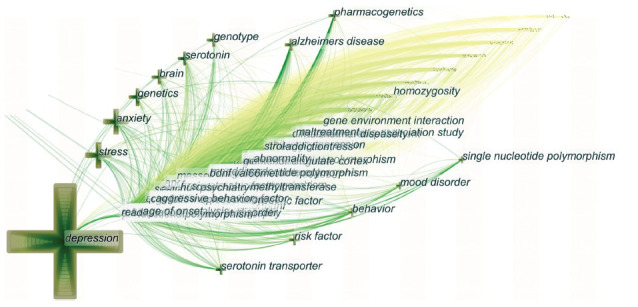
Timezone view of keyword co-occurrences in articles on depression-related SNP research from 1998 to 2021. The map shows nodes that represent keywords and the chronological order of keyword occurrences. The size of the nodes varies in direct proportion to how frequently certain keywords appear.

An analysis of the keywords revealed that several commonly used antidepressants, such as “citalopram”, “fluoxetine”, “paroxetine”, “escitalopram”, “venlafaxine”, “fluvoxamine”, “sertraline”, and “mirtazapine”, were among the top 10 related drugs studied in this field. The top 10 genes and receptors researched in the field, according to the analysis, were “5-hydroxytryptamine (5-HT)”, “*BDNF*”, “*COMT*”, “*FKBP5*”, “glucocorticoid receptor (GR)”, “*BDNF* val66met”, “*SLC6A4*”, “*CYP2D6*”, and “*CLOCK*” (Fig. **3C**).

### 3.7 Analysis of Burst Detection

**Fig. (6):**
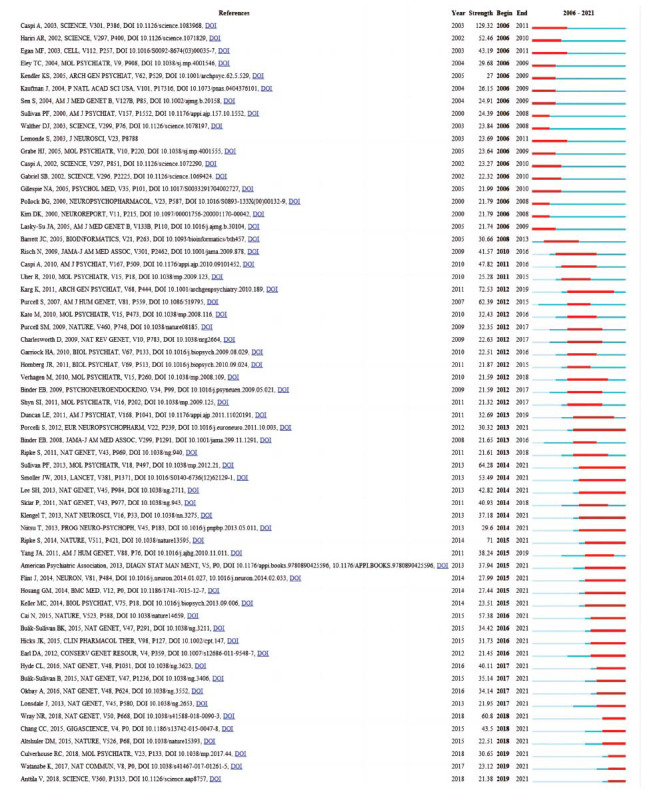
The strongest burst of citations in the literature on SNP research in the field of depression from 1998 to 2021. The intensity values reflect the frequency of citations. Red bars indicate frequently cited citations; green bars indicate rarely cited citations.

Through references with citation bursts, commonly cited literature throughout time can show how a subject of knowledge has developed. There were 61 references in total that had numerous significant citation bursts (Fig. **6**). The literature on the citation explosion first appeared in 2003 [37], and the most recent citation explosion literature first appeared in 2018 [38]. The strongest outburst (intensity: 129.32) occurred in 2006, appearing in an article from 2003. A burst of 25 references persisted until 2021 [39-42]. Burst keywords can be used to anticipate emerging subjects in particular fields of study. The top 41 keywords with the most powerful citation bursts are displayed in Fig. (**7**). The strongest outbreak word from 2006 to 2021 was “promoter polymorphism” (intensity: 29.59), which was followed by “major depression” (intensity: 23.18) and “affective disorder” (intensity: 22.45). The 2019 burst keywords, which include “*CYP2D6*”, “older adult”, “DNA methylation”, “polygenic risk score”, and “loci”, reflect current research trends. The explosion of additional keywords can also be utilized to examine how the study has changed over time. For instance, the original mouse brain is now being studied more in light of the individual variations in the human body. The findings also suggest that the precise pathogenesis of depression has been unclear in recent years, that SSRIs continue to be the most commonly prescribed medication, and that pathways connected to 5-HT will continue to be the main focus of this field of study [43, 44].

## 4 DISCUSSION

This is the first study to use bibliometric and visual analysis methods to investigate SNP in the field of depression. In order to present a thorough overview of the hotspots and trends in research on SNP in the field of depression during the past 23 years, we evaluated 10,043 papers from the WoSCC. The analysis of this study indicates that there has been a sharp increase in publications since 2006 and that there is active international collaboration in this area. The most influential countries, institutions, and academics are the United States, King's College London, and Caspi Avshalom, respectively. The most productive and frequently cited journals are the Journal of Affective Disorders and Biological Psychiatry, respectively. The largest topic cluster, “*BDNF*”, which represents significant research foundations in the field, is the focus of the research. Since 2006, the research hotspots in the field have been “*5-HTTLPR*” and “*BDNF*”. The most recent developments in the field of study are also reflected in the words “Alzheimer's illness”, “bipolar disorder”, “pharmacogenomics”, and “inbreeding depression”. Our findings thus offer valuable information and new perspectives for the investigation of SNP in depression. These findings help academics select appropriate journals and collaborators, understand the research status of other researchers in the field, and advance their research through our timely review and analysis of hotspots and research trends.

According to the findings of the collaborative network analysis, the United States has a clear advantage over other nations and regions in this regard, most likely as a result of its stronger economy and higher spending on scientific research. For instance, since 1983, the United States has conducted a number of studies on single nucleotide polymorphisms [45-48], which has positively impacted the quantity and caliber of following research in this area. It is significant to mention that England, the second-most productive nation, has an equal amount of impact in this area as the US. King's University London is a significant participant in the institution; the most frequently mentioned article was conducted by Caspi Avshalom, a professor at that university, and it was published in 2003 [25]. In this field, there is indeed close international scientific collaboration. Caspi Avshalom, for instance, is a research scholar at Duke University, which conducts roughly 29.7% of the college's research in the fields of medicine, pharmacy, and genetics. Caspi Avshalom is a leading expert in the disciplines of epigenetics, child abuse, multifactorial inheritance, summary statistics, and single nucleotide polymorphism. He was the first investigator to propose the hypothesis that genetic polymorphisms in the promoter region of the 5-hydroxytryptamine transporter protein (5-HTTLPR) are associated with a higher risk of depression in individuals exposed only to stressful situations. We advise scholars to concentrate on Caspi Avshalom's SNP-related representative research [49-51]. Furthermore, as this author focuses more on the relationship between attention deficit hyperactivity disorder, involutional depression, and genetic polymorphisms, the topic of the Lesch Klaus-Peter study is more firmly related to the field of this study [52-54]. The study by Lesch Klaus-Peter provides trends in the association between SNPs and mental disorders. Kendler Kenneth S. has studied as many as 100 themes, distinguishing himself from the other two authors by focusing more on the field of genomics. It is the perfect resource for scholars to understand information about genetics in this field because it uses terms like SNP, genotype-environment interaction, and multifactorial inheritance [55-57].

In the course of our research, we discovered that the top ten most active journals published roughly one-fifth (24.11%) of the publications in SNP research in the area of depression. However, overall, the distribution of the literature among journals was generally dispersed, likely as a result of the varied research directions, involving neuroscience, psychiatry, pharmacology/pharmacology, genomics, and epigenetics. As a result, there are numerous journals available for researchers to pick from in this field of study; however, choosing the best publication may be challenging due to how well each journal fits the research topic [58]. The outcomes of journals and co-cited journals could help researchers who study single nucleotide polymorphisms in depression. In addition, the top 15 most active journals and co-cited journals are only 40% consistent, so researchers can choose the appropriate journal based on the practical study and theme, and citing some authoritative journal articles in co-cited journals will give more credibility to the study.

The study by Wu *et al.*, published in Science in 2003 [25], reported that functional polymorphisms in the promoter region of the serotonin transporter (5-HTT) gene may be a significant factor in the regulation of stressful events and the generation of depression in various individuals, demonstrating that people's responses to their environments vary. This study was the first of the top 10 co-cited articles, according to the analysis of the literature co-citation network. The second-most cited article is Neil Risch's study from the Jama-Journal of the American Medical Association [59], in which the authors discuss the interaction between serotonin transporter protein genotypes and stressful life events associated with depression through a meta-analysis and also make reference to the aforementioned article of Caspi Avshalom. The theme cluster #1, “*5-HTTLPR*”, is related to nine of the top 15 highly referenced articles, and these publications serve as a significant research foundation in this field.

There was one of the strongest bursts of citations in 2006, and the strongest burst of literature was also the article by Caspi Avshalom published in Science [25]. An article published by Porcelli *et al.* in the 2012 issue of European Neuropsychopharmacology has been cited since 2013 and is still ongoing [60]. Their findings that the 5-HT transporter gene promoter polymorphism (5-HTTLPR) may function as a predictor of antidepressant drug response differ from those of previous studies. Their findings, however, imply that ethnic distinctions may be the cause and that *5-HTTLPR* may predict antidepressant response and remission in Caucasians but not in Asians. The most recent burst of citations occurred in 2019, the strongest of which was an article published by Culverhouse *et al.* in the journal Molecular Psychiatry in 2018 [61]. If the hypothesis of an interaction between stress and the S allele of *5-HTTLPR* that increases the risk of depression is correct, it may only be in stressed individuals and is not broadly generalized, according to study mate analytical techniques for a large-scale study on the results obtained by Caspi Avshalom in 2003.

We divided the keywords into two categories based on the results of the keyword analysis: genes/receptors and drug. Among the top 10 genes/receptors, “5-HT” appeared the most frequently. 5-HT neurotransmitters are considered crucial in regulating emotion and behavior; 5-HT has been the subject of research in the field of depression [62, 63]. With the development of genomics, scientists have started to focus on genes encoding 5-HT, where 5-HT transporter genes and 5-HT receptor genes have been demonstrated to be associated with depression and the efficacy of antidepressant drugs [64, 65]. For example, in adults with depressive symptoms, there is a correlation between the *5-HTT* and childhood adversity, whereas carriers of the S allele of the *5-HTT* are less likely to have depressive symptoms [66]. A study on *5-HT1A* (*HTR1A*) found that rs6295 promoter polymorphisms were associated with anxiety phenotypes, and loss and blockade of *5-HT1A* heterogeneous receptors were observed to increase anxiety and block SSRI efficacy in animal models [67]. In addition, the top 10 drugs in the keyword list belong to Selective Serotonin Reuptake Inhibitor. In the field of depression, 5-HT-related genes (*e.g*., *HTR1A*, *HTR2A*, and *SLC6A4*) and their SNPs are also the predominantly researched pharmacodynamic genes [68-70]. Solute carrier family 6 member 4 * (SLC6A4)* , also known as the 5-hydroxytryptamine transporter protein gene, was the first gene with an SNP to be reported [71]. *SLC6A4* is also one of the most studied target genes in neurobiological genetics association [72-76]. The *SLC6A4* polymorphism has also been associated with SSRI response to depression, and depressed patients with the *SLC6A4* rs25531 LL genotype have been proven to respond better to fluoxetine than patients with other genotypes in the Indian population [77]. Furthermore, *SLC6A4* gene polymorphisms have been associated with the pathogenesis and risk of depression [78-80].

In the burst analysis of keywords, “*CYP2D6*” started to appear in 2009 and is still in a burst from 2019 to the present. Genetic variation in drug-metabolizing enzymes is mainly affected by cytochrome P450 (CYP) enzymes [81], so individual differences caused by polymorphisms in CYP family metabolizing enzyme genes affect the efficacy of antidepressant drugs and the occurrence of adverse effects, and this phenomenon has also received attention from research scholars [82-85]. Among CYP metabolizing enzyme genes, the most studied genes are mainly *CYP2D6*, *CYP2C19*, and *CYP2C9* [86]. Nowadays, most of the metabolic enzyme genes involved in antidepressants in the market are CYP450 enzyme genes, with up to 25% of antidepressants and antipsychotics metabolized by * CYP2D6* [87]. *CYP2D6* has the highest degree of SNPs [88, 89], with the most significant variants including *2, *3, *4, *5, *10, *17, and *41. CYP2D6 is involved in the metabolism of 5-HT in the brain, which is linked to psychiatric disorders, as well as the synthesis of serotonin and dopamine [90]. Among these alleles, *10 is a mutation with a high allele frequency in Asian populations [91]. A study on patients with recurrent depression found that an SNP in *CYP2D6* was associated with the efficacy of the antidepressant duloxetine [92]. The *CYP2D6* genotype has also been shown to have a possible impact on the length of hospitalization and risk of hospitalization in patients with major depressive disorder, thereby increasing the disease and financial burden on patients [93]. Unfortunately, SSRIs are currently ineffective in 60-70% of patients [94-97]. With the prevalence of the concept of precision individualized drug therapy, the study on drug-metabolizing enzymes and their SNPs in association with drugs will receive more attention.

Besides the metabolic enzyme genes, we also focus on the second “*BDNF*” and third “*COMT*” genes. BDNF is a neuron that acts on the hippocampus, cerebral cortex, and cerebellum [98]. It can play an essential role in the pathogenesis of depression by modulating neuronal and molecular proteins that affect the transmission of serotonin and dopamine [99]. The neurotrophic factor hypothesis indicates that a decrease in * BDNF* in the cortical and limbic regions of the brain is associated with depression [100-102]. Among the SNP studies on *BDNF* associated with depression, the most reported and studied is rs6265 (Val66Met) [103-105], and some studies have shown that the *BDNF* Val66Met polymorphism correlates with serum BDNF levels and can be used as a predictor of major depressive disorder [106]. The methylase catechin-o-methyltransferase (COMT), which is responsible for degrading catecholamine, dopamine, and epinephrine, is encoded by *COMT* [107]. Due to its ability to control the neurotransmitter dopamine [108], * COMT* is crucial in the investigation of psychiatric disorders like depression [109]. More than 4,000 SNPs have been identified in *COMT* to date [110], with Val158Met (rs4680) being one of the most common functional SNPs used in *COMT* for depression research [111, 112]. *COMT* rs4680 has been revealed in several research studies to correlate with the risk of developing depression and pathogenesis [113, 114]. Furthermore, rs4680 has been reported to correlate with fluvoxamine efficacy [115], while *COMT* rs25531 correlated with MDD response to fluoxetine treatment [116]. The above analysis indicates that the genes and its SNPs being studied in SNP studies on depression are involved not only in the pathogenesis of depression but also in the study of multiple aspects of patients' risk of developing depression and the efficacy and adverse effects of antidepressant drugs. These genes and polymorphisms are the focus of attention and can contribute to field as a potential indicator for the treatment, diagnosis, and prevention of depression.

## 5 LIMITATION

This study had some limitations. The primary search data were sourced solely from the WoSCC database, mainly because the data and research provided by this database are widely accepted to have the highest quality and reliability, and as such, it is the first choice for bibliometric analysis. However, this can also lead to some elements of bias and incompleteness in the results. The analysis of the authors and co-authors of the literature in this study may be somewhat flawed because the Citespace software does not fully distinguish between the names and abbreviations of the various authors. The amount of data analyzed in this study was large enough to consider that the results of this study are reflective of the general trend and current situation in the field. As a result, this study can serve as a reference framework for researchers investigating the relationship between SNPs and depression.

## CONCLUSION

This study conducted a bibliometric and visual analysis of the literature on SNPs and depression from 1998 to 2021. The study results identified research hotspots, key countries and institutions, and research directions in this field over the past 23 years. Since 2006, the number of publications has maintained relatively steady growth. Overall, there is close international scientific collaboration in this field, with authors, such as Caspi Avshalom, Lesch Klaus-Peter, and Kendler Kenneth S, further laying a solid research foundation. Due to the diversity of research directions, researchers have a multitude of journals to choose from. Nevertheless, the pathogenesis of depression is still unclear, so “5-HT” is still the focus of current research, which also reflects an essential research base in this field. This study explored the association between SNPs and depression and found that, at present, various genes and SNPs related to the etiology and mechanism of depression are more frequently studied in genomics. “*CYP2D6*” has been in the spotlight since its emergence in 2009 and since then, it has become a research hotspot. However, “*BDNF*”, “*COMT*”, ” older adults”, “loci” and “DNA methylation” are also among the new frontier of research and some of them are currently in the process of exploration. Therefore, we hope to contribute to the development of this field by providing a timely review and analysis.

## Figures and Tables

**Fig. (4):**
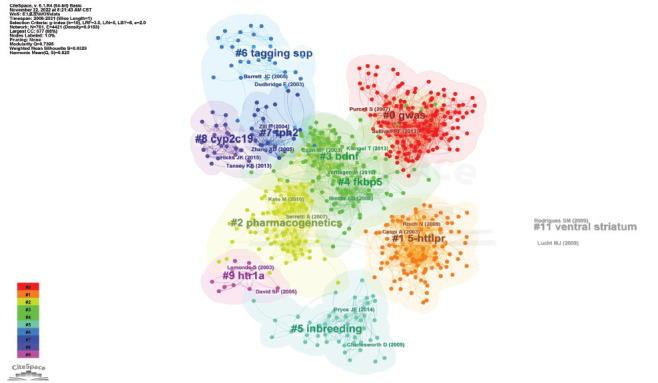
Clustering visualization plot for reference co-citation network analysis of the publications on SNP in the field of depression from 1998 to 2021.

**Fig. (7):**
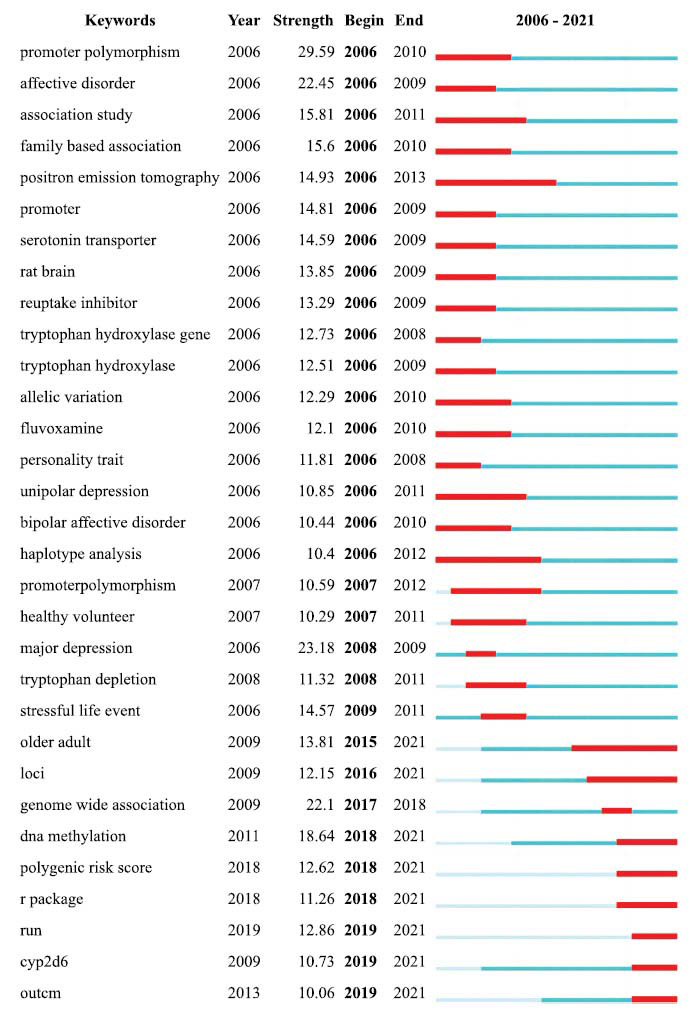
The keywords with the strongest outbreak of citations in publications on SNP research in the field of depression published from 1998 to 2021.

**Table 1:** The top 15 countries/regions, institutions, and authors in terms of publications.

**Items**	**Publication**		
	**Ranking**	**Name**	**Count**
Country/Region	1	America	3477
	2	England	1301
	3	Germany	1096
	4	China	1009
	5	Australia	711
	6	Canada	689
	7	Italy	684
	8	Netherlands	627
	9	Japan	482
	10	France	433
	11	Sweden	370
	12	Spain	368
	13	South Korea	307
	14	Brazil	297
	15	Switzerland	292
Institution	1	King's College London	314
	2	University of Toronto	213
	3	Duke University	198
	4	Karolinska Institute	195
	5	University of Pittsburgh	191
	6	Univ Bonn Germany	182
	7	University of Bologna	181
	8	Max Planck Institute Psychiat	172
	9	National Institute of Mental Health	157
	10	Columbia University	154
	11	Harvard University	153
	12	Vrije University Amsterdam	150
	13	Yale University	146
	14	University of Melbourne	141
	15	Massachusetts General Hospital	133
Author	1	Serretti Alessandro	150
	2	Rietschel Marcella	85
	3	Domschke Katharina	65
	4	Boomsma Dorret I	58
	5	Binder Elisabeth B	56
	6	Baune Bernhard T	50
	7	Holsboer Florian	49
	8	Maier Wolfgang	48
	9	Iwata Nakao	47
	10	Arolt Volker	47
	11	Mors Ole	44
	12	Fabbri Chiara	44
	13	Mcguffin Peter	43
	14	Breen Gerome	42
	15	Gelernter Joel	41

**Table 2:** The top 15 journals and co-cited journals for SNP research in the depression field.

**Item**	**Ranking**	**Name**	**Country**	**Counts**	**IF**
Jounrnal	1	Journal of Affective Disorders	Netherlands	324	6.533
	2	Plos One	USA	283	3.752
	3	American Journal of Medical Genetics Part B Neuropsychiatric Genetics	USA	234	3.358
	4	Translational Psychiatry	England	183	7.989
	5	Molecular Psychiatry	England	176	13.437
	6	Biological Psychiatry	USA	158	12.810
	7	Progress in Neuro-Psychopharmacology and Biological Psychiatry	England	153	5.201
	8	Psychiatry Research	Netherlands	133	11.225
	9	Neuroscience Letters	Netherlands	129	3.197
	10	Journal of Psychiatric Research	England	123	5.250
	11	Psychoneuroendocrinology	England	114	4.693
	12	Neuropsychopharmacology	England	113	8.294
	13	Psychiatric Genetics	USA	111	2.574
	14	Genes Brain And Behavior	Denmark	106	3.708
	15	International Journal of Neuropsychopharmacology	England	81	5.678
Co-cited Journal	1	Biological Psychiatry	USA	5470	12.810
	2	Molecular Psychiatry	England	5249	13.437
	3	Archives of General Psychiatry	USA	4634	/
	4	American Journal of Psychiatry	USA	4590	19.242
	5	Science	USA	4463	63.714
	6	Proceedings of The National Academy of Sciences of The United States of America	USA	4339	12.779
	7	Neuropsychopharmacology	England	3714	8.294
	8	Journal of Affective Disorders	Netherlands	3424	6.533
	9	PloS One	USA	3341	3.752
	10	Nature	England	3323	69.504
	11	American Journal of Medical Genetics Part B Neuropsychiatric Genetics	USA	3254	3.358
	12	American Journal of Human Genetics	USA	2927	11.043
	13	Journal of Neuroscience	USA	2905	6.167
	14	Psychological Medicine	USA	2412	10.592
	15	Nature Genetics	England	2408	41.307

**Table 3:** The top 10 co-cited authors with the most citations.

**Ranking**	**Times Cited**	**Author**	**Institution (Country)**	**Major Research Field**	**H-Index**
1	1385	Caspi Avshalom	Duke University (United States) King’s College London, London (United Kingdom)	Neurosciences/Neurology, Psychiatry, Genetics/Heredity	149
2	1161	Lesch Klaus-Peter	Maastricht University and Neuroplast BV (Netherlands) University of Würzburg (Germany)	Neurosciences/Neurology, Psychiatry, Genetics/Heredity, Pharmacology/Pharmacy	110
3	1045	Kendler Kennet S	Virginia Commonwealth University (United States)	Neurosciences/Neurology, Psychiatry, Genetics/Heredity, Pharmacology/Pharmacy	156
4	872	Serretti Alessandro	University of Bologna (Italy)	Neurosciences/Neurology, Psychiatry, Genetics/Heredity, Pharmacology/Pharmacy	72
5	762	Kessler Ronald C	Harvard Medical School (United States)	Neurosciences/Neurology, Psychiatry, Genetics/Heredity, Pharmacology/Pharmacy	227
6	614	Egan Michael F	Merck Research Laboratories, Merck (United States)	Neurosciences/Neurology, Psychiatry, Genetics/Heredity, Pharmacology/Pharmacy	69
7	478	Sullivan Patrick F	Karolinska Institutet (Sweden) University of North Carolina (United States)	Neurosciences/Neurology, Psychiatry, Genetics/Heredity, Pharmacology/Pharmacy	103
8	454	Purcell Shaun	Harvard Medical School, Harvard University (United States)	Neurosciences/Neurology, Psychiatry, Genetics/Heredity, Pharmacology/Pharmacy	103
9	450	Hariri Ahmad R	Duke University (United States)	Neurosciences/Neurology, Psychiatry, Genetics/Heredity, Pharmacology/Pharmacy	68
10	408	Heils Armin	Rheinische Friedrich-Wilhelms-University of Bonn (Germany)	Neurosciences/Neurology, Psychiatry, Genetics/Heredity, Pharmacology/Pharmacy	37

**Table 4:** The top15 co-cited references related to SNP research in the depression field between 1998 and 2021.

**Ranking**	**Cited ** **Number**	**Year**	**Title of Article**	**Author**	**Journal**	**IF**
1	575	2003	Influence of life stress on depression: moderation by a polymorphism in the 5-HTT gene	Caspi Avshalom	Science	63.714
2	370	2009	Interaction between the serotonin transporter gene (5-HTTLPR), stressful life events, and risk of depression: a meta-analysis	Risch Neil	Jama-Journal of The American Medical Association	157.355
3	343	2005	Haploview: analysis and visualization of LD and haplotype maps	Barrett J. C.	Bioinformatics	6.931
4	333	2011	The serotonin transporter promoter variant (5-HTTLPR), stress, and depression meta-analysis revisited: evidence of genetic moderation	Karg Katja	Archives of General Psychiatry	/
5	289	2007	PLINK: a tool set for whole-genome association and population-based linkage analyses	Purcell Shaun	American Journal of Human Genetics	11.043
6	285	2006	Serotonin transporter promoter gain-of-function genotypes are linked to obsessive-compulsive disorder	Hu Xian-Zhang	American Journal of Human Genetics	11.043
7	230	2005	5-HTTLPR polymorphism impacts human cingulate-amygdala interactions: a genetic susceptibility mechanism for depression	Pezawas Lukas	Nature Neuroscience	28.771
8	222	2005	The interaction of stressful life events and a serotonin transporter polymorphism in the prediction of episodes of major depression: a replication	Kendler Kenneth S	Archives of General Psychiatry	/
9	216	2010	Genetic sensitivity to the environment: the case of the serotonin transporter gene and its implications for studying complex diseases and traits	Caspi Avshalom	American Journal of Psychiatry	19.242
10	212	2013	A mega-analysis of genome-wide association studies for major depressive disorder	Major Depressive Disorder Working Group of the Psychiatric GWAS Consortium	Molecular Psychiatry	13.437
11	196	2003	The BDNF val66met polymorphism affects activity-dependent secretion of BDNF and Human Memory and Hippocampal Function	Egan Michael F	Cell	66.849
12	179	2014	Biological insights from 108 schizophrenia-associated genetic loci	Schizophrenia Working Group of the Psychiatric Genomics Consortium	Nature	69.504
13	167	2004	Gene-environment interaction analysis of serotonin system markers with adolescent depression	Eley Thalia C	Molecular Psychiatry	13.437
14	166	2002	Serotonin transporter genetic variation and the response of the human amygdala	Hariri Ahmad R	Science	63.714
15	159	2013	Identification of risk loci with shared effects on five major psychiatric disorders: a genome-wide analysis	Cross-Disorder Group of the Psychiatric Genomics Consortium	Lancet	202.731

**Table 5:** The top 10 keywords in terms of frequency for SNP research in the depression field.

**Item**	**Ranking**	**Frequency**	**Keyword**
Total	1	3190	Depression
	2	2069	Polymorphism
	3	1920	Association
	4	1429	Major depression
	5	931	Major depressive disorder
	6	908	Gene
	7	877	Schizophrenia
	8	848	Bipolar disorder
	9	834	Stress
	10	796	Anxiety
Drug	1	246	Citalopram
	2	209	Fluoxetine
	3	192	Paroxetine
	4	148	Escitalopram
	5	111	Venlafaxine
	6	103	Fluvoxamine
	7	91	Sertraline
	8	78	SSRI
	9	52	Mirtazapine
	10	16	Morphine
Gene/Receptor	1	1347	*5-HT*
	2	761	*BDNF*
	3	274	*COMT*
	4	139	Glucocorticoid receptor
	5	116	Monoamine oxidase
	6	110	BDNF val66met polymorphism
	7	101	*FKBP5*
	8	89	*SLC6A4*
	9	80	*CYP2D6*
	10	65	*CLOCK*

## Data Availability

Not applicable.

## LIST OF ABBREVIATIONS

5-HTTLPR: 5-hydroxytryptamine Transporter Protein
GR: Glucocorticoid Receptor
SNP: Single Nucleotide Polymorphism
SSRI: Selective Serotonin Reuptake Inhibitor
